# A Rare Presentation of Duplicate Superior Vena Cava Precipitating Premature Atrial Contractions: A Case Report and a Review of the Literature

**DOI:** 10.7759/cureus.37791

**Published:** 2023-04-18

**Authors:** Iyad Y Idries, Avtar Sur, Ruchi Yadav, Vivek Yadav, Vijay Jaswani, Mohammed Zaman

**Affiliations:** 1 Internal Medicine, Brookdale University Hospital Medical Center, New York City, USA; 2 Pulmonary and Critical Care, State University of New York Downstate Health Sciences University, New York City, USA; 3 Interventional Radiology, Brookdale University Hospital Medical Center, New York City, USA; 4 Critical Care Medicine, Brookdale University Hospital Medical Center, New York City, USA

**Keywords:** dilated right atrium, triple lumen catheter, premature atrial complexes, persistent left svc, dilated coronary sinus, duplicate superior vena cava

## Abstract

Millions of central lines are placed each year worldwide for life-saving measures. We present a case of left internal jugular (IJ) triple lumen catheter (TLC) placement for life-saving vasopressors, which appeared to be in the left mediastinum after a confirmed chest X-ray. After correlation with a previous MRI of the heart with and without contrast, duplication of the superior vena cava (SVC), also known as persistent left SVC (PLSVC), was discovered. PLSVC often causes no symptoms to affected individuals and is usually first found as an incidental finding discovered during thoracic surgeries, cardiovascular interventional procedures, and central line insertions. Placement of TLC or central venous catheter (CVC) can be challenging in such patients and may lead to life-threatening complications such as severe arrhythmias, cardiogenic shock, pneumothorax, and tamponade. Knowing such anomalies can prevent unnecessary catheter removal and help determine the origin of some arrhythmias and dilated heart chambers in these patients.

## Introduction

Superior vena cava (SVC) duplication occurs when a right SVC coexists with a persistent left SVC (PLSVC). SVC duplication is an infrequent entity present in 0.3% of the general population [1]. A left-sided SVC coexists with a right-sided physiologic SVC because of differences in the embryonic thoracic venous system's development. Embryologically, when the left cardinal vein, which usually regresses and becomes the Ligament of Marshall, fails to do so, it persists as the PLSVC [2]. A persistent right SVC accompanies 90% of PLSVCs, while in 10% of cases, PLSVC is an isolated defect [3].

When the catheter does not cross the midline on imaging studies during a left-sided endovascular procedure, SVC duplication can create confusion and concern even though most patients are asymptomatic. This condition is associated with cardiac anomalies such as atrial septal defect (ASD), ventricular septal defect (VSD), and, more rarely, coronary sinus ASD, an atypical form of ASD in which the left SVC drains directly into the right atrium. Coronary sinus ASD comprises 1% of all ASD causes, making it the rarest of them all [4].

## Case presentation

We report a case of a 65-year-old female admitted to the ICU for hypercapnic hypoxic respiratory failure requiring intubation. Her admission was complicated by an acute GI bleed and severe hypotension requiring immediate fluid resuscitation and vasopressors. Initially, a right internal jugular (IJ) triple lumen catheter (TLC) was placed, which was confirmed. On the seventh day of right IJ, a left IJ TLC was indicated per institute protocol, and a left-sided TLC was attempted.

**Figure 1:**
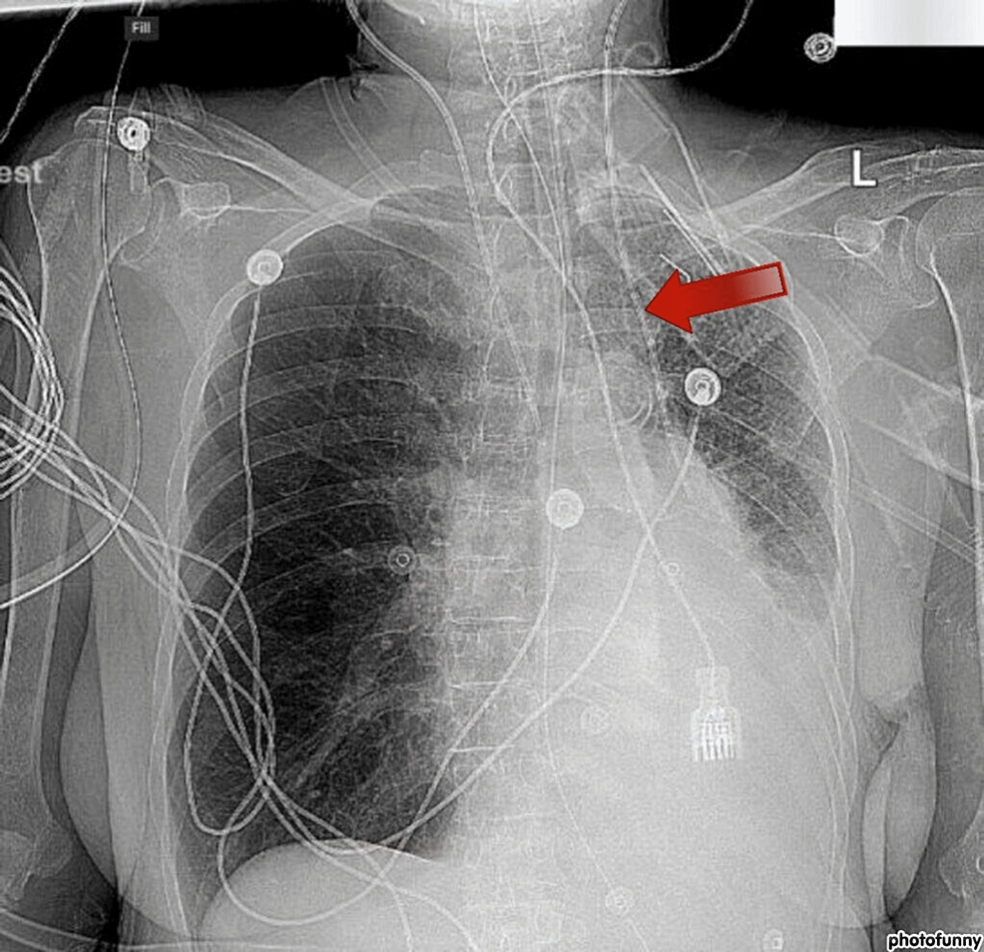
Left triple lumen catheter placement noted on the left side of the mediastinum

The left IJ was accessed as per the accepted technique during the procedure. The guidewire was removed, and normal blood flow from all three ports was observed. A venous blood gas (VBG) was collected, which showed typical values for a venous insertion; however, a chest X-ray showed unusual caudal movement on the left side without crossing the midline (Figure 1).

**Figure 2:**
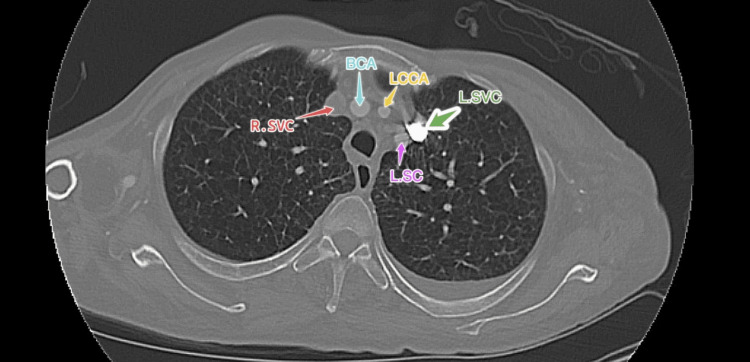
Computed tomography angiography scan demonstrating duplication of superior vena cave R.SVC - right superior vena cava; BCA - brachiocephalic artery; LCCA - left common carotid artery; LSC - left subclavian artery; L.SVC - left subclavian artery

**Figure 3:**
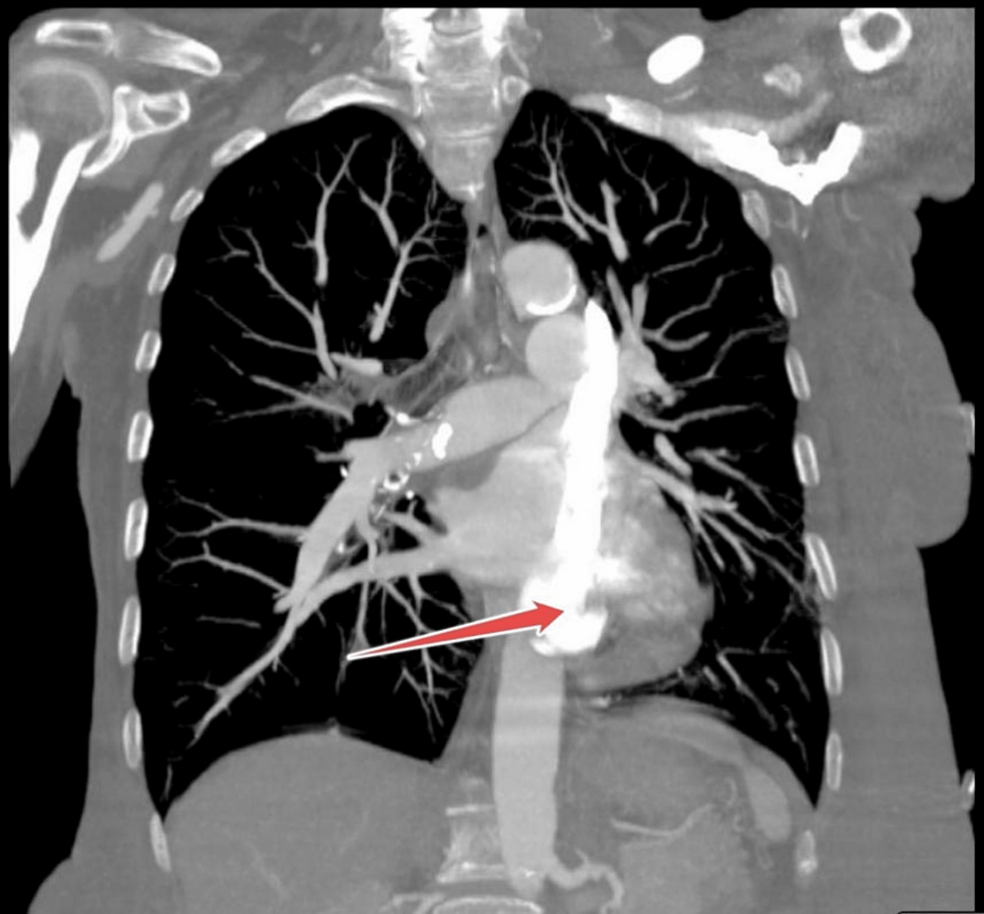
Computed tomography angiography chest indicating the left SVC entering the right atrium (the red arrow) SVC - superior vena cava

**Figure 4:**
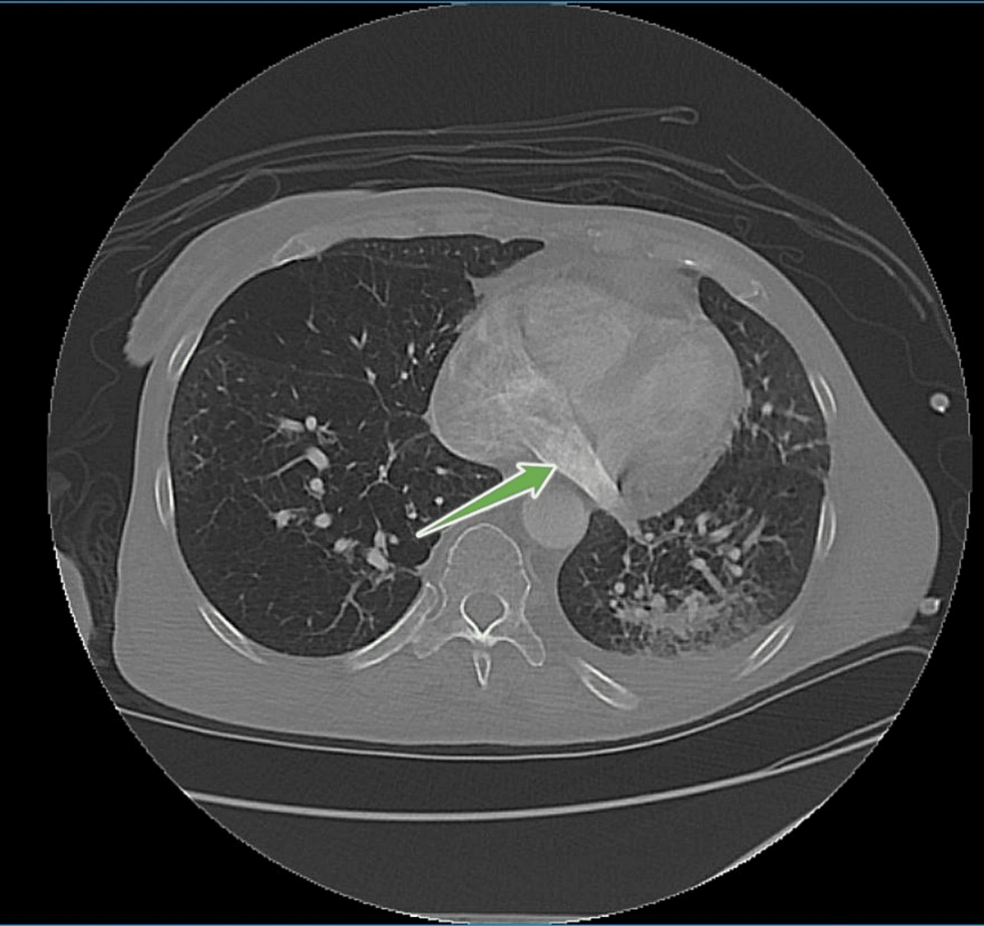
Computed tomography angiography chest demonstrating contrast passing via the left SVC into the coronary sinus and then to the right atrium (green arrow) SVC - superior vena cava

A full review of past imaging revealed that seven years ago, the patient had a cardiac magnetic resonance imaging with and without contrast performed, showing duplication of the superior vena cave, confirmed on a computed tomography angiography (CTA) scan done after admission to rule out pulmonary embolism (PE), left antecubital vein was used for contrast administration which made the LSVC more visible (Figures 2-4).

**Figure 5:**
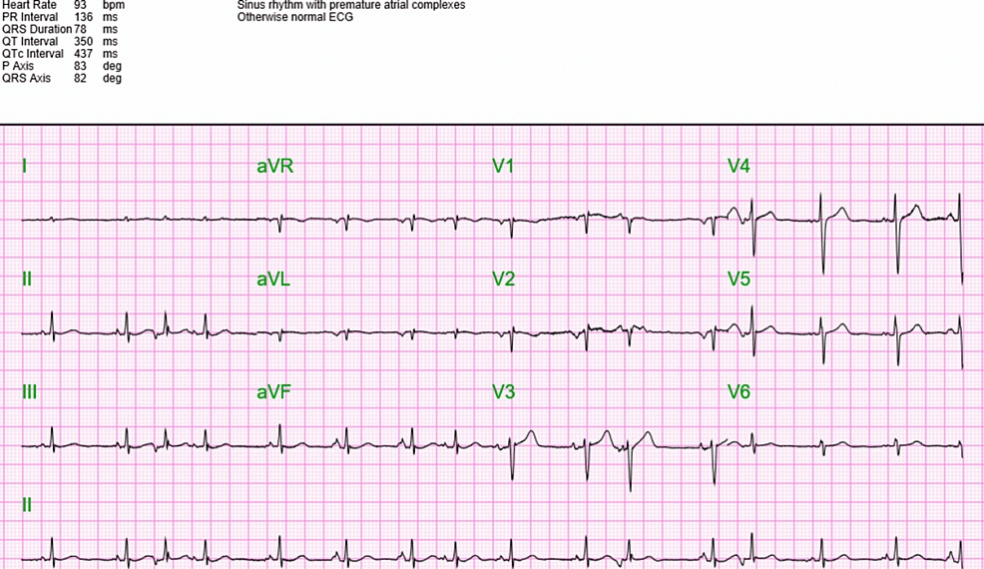
EKG demonstrating premature atrial complexes (PACs)

Eight years prior, a transthoracic echo (TTE) with bubble study showed a severely dilated right ventricle (RV) with prominent coronary sinus. A bubble study on TTE showed the passage of agitated saline into the left ventricle (LV) and persistent SVC when injected through the left arm. After correlating the bubble study with cardiac CT, the patient appears to have a coronary sinus ASD variant. In addition, the dilated ventricle can explain the patient's persistent premature atrial complexes (PACs), as demonstrated on the patient's EKG (Figure 5). 

## Discussion

The right SVC drains blood from the upper body and is where TLCs are placed. The average length of the SVC is about seven centimeters, emptying into the right atrium [5]. Embryologically, the umbilical, vitelline, and cardinal veins develop and evolve during the first two months of fetal life to form the various venous thoracic systems. The upper limbs, neck, upper torso, and head are drained via the bilateral anterior cardinal veins, which drain through the bilateral posterior cardinal veins, also known as post-cardinal veins [6]. The commonest normal variant develops when the right anterior cardinal and common cardinal veins form the SVC, and the left anterior cardinal vein regresses to become the ligament of Marshall. A double SVC results when both the right and left anterior cardinal veins persist due to failure of degeneration/involution of the left anterior cardinal vein proximal to the brachiocephalic anastomosis [7-9]. Only 0.3% of the general population have the anomaly of duplicate SVC, and 4.5% of them have associated cardiac abnormalities [1,10].

Most left SVC patients are asymptomatic and do not require treatment since the left SVC drains into the right atrium. However, since the SVC drains directly into the right coronary sinus (Figure 4), the placement of catheters might lead to irritation of the coronary sinus and result in hypotension, arrhythmia, myocardial ischemia, and cardiac arrest. In addition, in a minority of these patients, where the SVC drains into the left atrium, such a catheter can increase the risk of systemic air emboli and right-sided heart failure [11].

**Figure 6:**
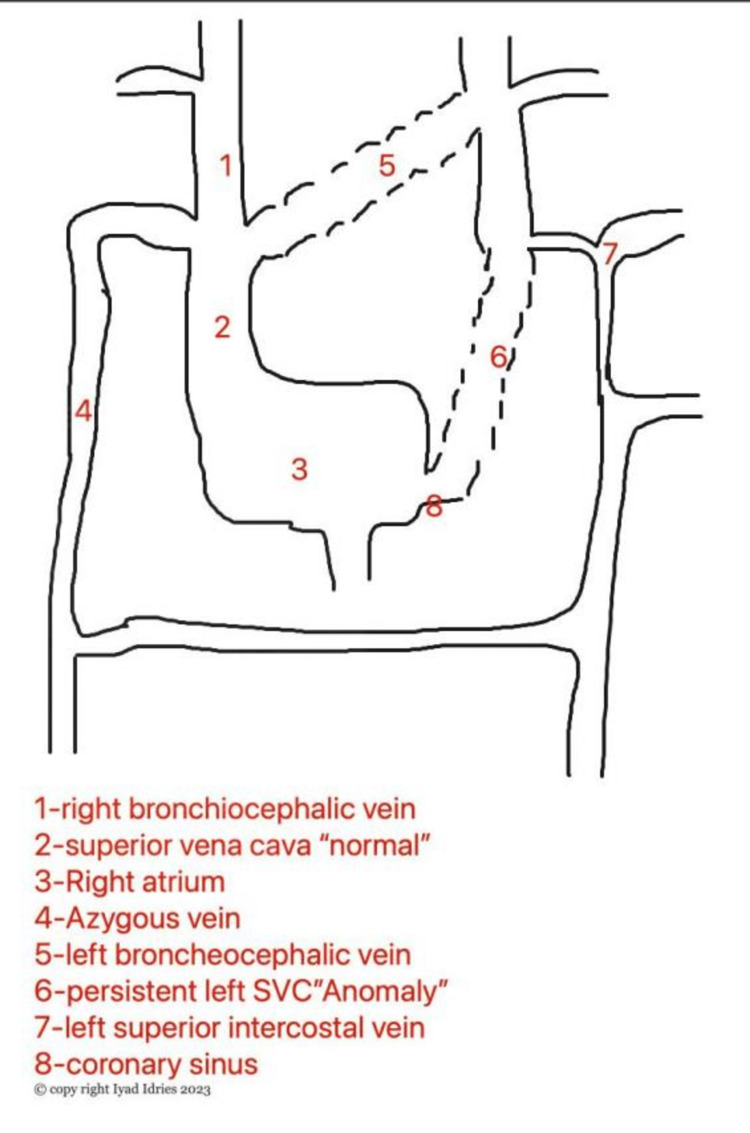
Embryological development of precardinal venous system SVC - superior vena cava

The PLSVC drains 80-90% through the coronary sinus into the right atrium, and in the remaining 10-20% of cases, it empties into the left atrium. In such cases, the PLSVC drains directly into the left atrium or via the unroofed coronary sinus, which results in a right-to-left shunt of clinical significance [12]. The coronary sinus is frequently dilated in cases of PLSVC with right atrial drainage, as seen in our patient. The atrioventricular node and Its bundle may be compressed because of this enlargement causing cardiac arrhythmias like atrial/ventricular fibrillation, left atrial compression, decreased cardiac output leading to cardiac symptoms of fatigue, decreased exercise tolerance, chest pain, palpitations, cyanosis, and syncope [13].

In clinicians involved in advanced central venous device placements, awareness of duplicate SVC or PLSVC is critical. Accessing the right side of the heart or the pulmonary vasculature is much more challenging via the left subclavian vein and can result in incorrect positioning of these devices. These complications of incorrect positioning are often seen in undiagnosed PLSVC and, when undetected, can further lead to more severe concerns like cardiogenic shock, tamponade, perforation, coronary sinus thrombosis, and arrhythmias [14,15]. In these cases, access to the right heart and coronary sinus should be obtained through the right subclavian vein, which is a more direct and safer route, given the anatomical variation.

Should there be a high suspicion of a persistent left SVC upon seeing the catheter taking an abnormal course on a chest X-ray. In that case, definitive imaging is recommended by cross-sectional computed tomography with angiography or MR angiogram of the chest, which is much safer than removing and reinserting a new catheter [6,16]. A PLSVC should be suspected whenever a catheter or guide wire inserted via the left subclavian vein is seen taking an unusual left-sided downward course on an X-ray chest. A dilated coronary sinus on echocardiography should raise the clinician's suspicion of duplicate SVC or PLSVC. Saline contrast or a bubble study on echocardiography should be used to confirm the diagnosis [17].

TLC CVC insertion is an invasive procedure that requires prior knowledge of the PLSVC existence. Angina, hypotension, and even heart perforation could result during CVC placement without fluoroscopy. In addition, CVC catheterization can be difficult and may lead to life-threatening complications such as severe arrhythmias, cardiogenic shock, pneumothorax, and tamponade in patients [18].

Knowing such anomalies can prevent unnecessary catheter removal and help determine the origin of some arrhythmias and dilated heart chambers in these patients that can be seen.

## Conclusions

Knowledge of variant vessel anomalies, such as duplicate SVC, can help appropriately manage these patients in the presence of a central catheter. It can also help determine the causality of the arrhythmias in some patients, preventing various complications.
